# A Preliminary X-ray Study of Murine Tnfaip8/Oxi-α

**DOI:** 10.3390/ijms15034523

**Published:** 2014-03-14

**Authors:** Daeun Lee, Mi-Sun Kim, Jimin Park, Gil-Ja Jhon, Jin H. Son, Dong Hae Shin

**Affiliations:** 1 College of Pharmacy and Graduate School of Pharmaceutical Sciences, Global Top5 Research Program, Ewha Womans University, Seoul 120-750, Korea; E-Mails: yidaeun89@gmail.com (D.L.); shfwk31@gmail.com (M.-S.K.); whitedakd@gmail.com (J.P.); hjson@ewha.ac.kr (J.H.S.); 2 Department of Chemistry and Nano Science, Global Top5 Research Program, Ewha Womans University, Seoul 120-750, Korea; E-Mail: gjjhon@ewha.ac.kr

**Keywords:** Parkinson’s disease, Oxi-α, oxidative stress, C165S mutant, X-ray crystallography

## Abstract

Tnfaip8/oxidative stress regulated gene-α (Oxi-α) is a novel protein expressed specifically in brain dopaminergic neurons and its over-expression has been reported to protect dopaminergic cells against OS-induced cell death. In this study, murine C165S mutant Tnfaip8/Oxi-α has been crystallized and X-ray data have been collected to 1.8 Å using synchrotron radiation. The crystal belonged to the primitive orthorhombic space group P2_1_2_1_2, with unit-cell parameters *a* = 66.9, *b* = 72.3, *c* = 93.5 Å. A full structural determination is under way in order to provide insights into the structure-function relationships of this protein.

## 1. Introduction

Parkinson’s disease (PD) is a degenerative disorder of the central nervous system. It is associated with a selective loss of the dopaminergic neurons in the midbrain area called substantia nigra pars compacta [1]. Since these neurons are essential for voluntary movements, their loss is associated with obvious symptoms such as tremor, rigidity, bradykinesia and postural instability. Oxidative stress (OS) plays a significant pathogenic role in the progress of PD [2]. Tnfaip8/oxidative stress regulated gene-α (Oxi-α) is a novel protein expressed specifically in brain dopaminergic neurons and participates in OS-induced signaling [3]. The over-expression of Tnfaip8/Oxi-α has been reported to protect dopaminergic cells against OS-induced cell death. On the contrary, the *Oxi*-α gene knockdown affected dopaminergic neuronal susceptibility to OS. Oxi-α exerts this dopaminergic cell protective role by reducing autophagic cell death that is induced by OS [4]. Over-expression of Tnfaip8/Oxi-α dramatically inhibited the accumulation of autophagic vacuoles induced by autophagy inducers and OS. Intriguingly, the protective action of Tnfaip8/Oxi-α involves the activation of mTOR kinase which is a key member of the classical mTOR-dependent pathway of autophagy [4]. Increased levels of Tnfaip8/Oxi-α activate mTOR kinase regardless of the presence of rapamycin by increasing mTOR phosphorylation through an unknown mechanism. Although the precise mechanism by which Tnfaip8/Oxi-α activates mTOR kinase is not clear, Tnfaip8/Oxi-α is the first molecule to activate mTOR kinase and subsequently suppress the accumulation of autophagic vacuoles.

In order to elucidate the structural and functional relationship of the Tnfaip8/Oxi-α family, we have initiated the determination of the three-dimensional structure of murine Tnfaip8/Oxi-α. There are four Tnfaip8/Oxi-α family members with ~54% sequence identity (Table 1). The crystal structure of Tnfaip8l2/Oxi-γ/TIPE2 has been published [5]. It belongs to all alpha proteins in SCOP [6] and shows a unique fold with a large hydrophobic central cavity. Tnfaip8l2 is essential for maintaining immune homeostasis [7] and plays a key role in a signal transduction pathway that links the inflammatory immune response to specific conditions after cerebral ischemia. Therefore, it is quite intriguing to elucidate common or unique structural properties of these families conferring diverse cellular functions. In this study, we report the cloning, overexpression, purification, crystallization and preliminary X-ray study of this enzyme.

## 2. Results and Discussion

### 2.1. Protein Expression and Purification

**Figure 1.**
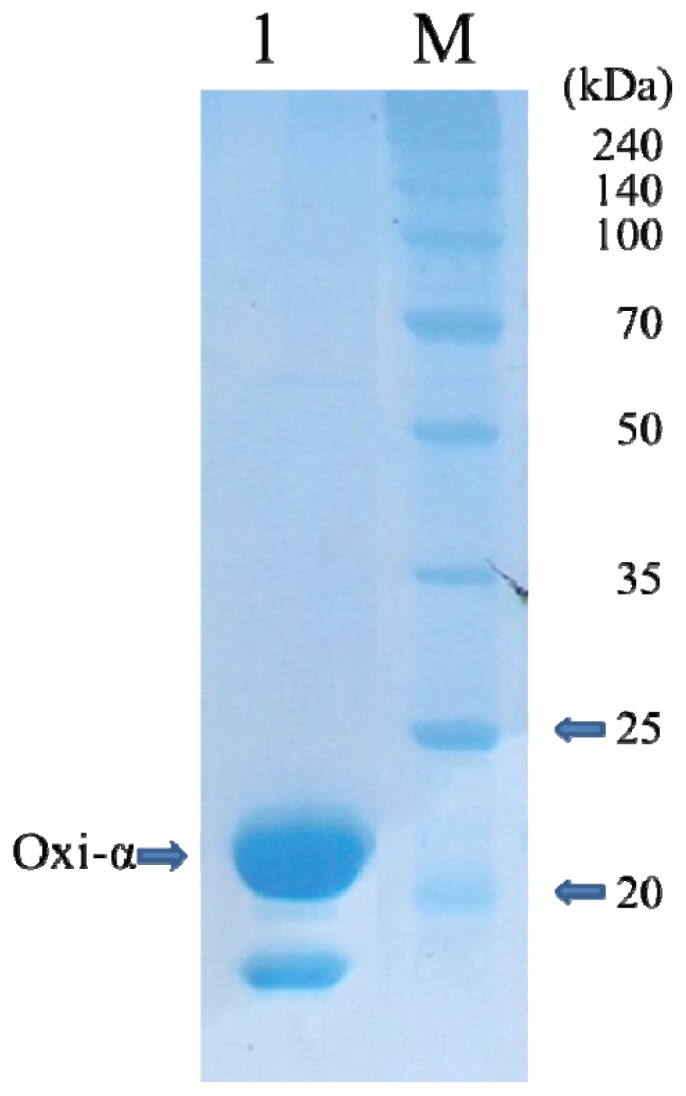
Coomassie-stained SDS-PAGE (12%) of C165S mutant Oxi-α. Lane 1, C165S mutant Oxi-α (1.1 mg); Lanes M, molecular-mass marker (labeled in kDa).

The purification of native murine Tnfaip8/Oxi-α was not successful due to its heavy precipitation. Therefore, the C165S mutant was designed to avoid the precipitation induced presumably by non-specific disulfide bond formation. This strategy was successful and the mutant Tnfaip8/Oxi-α was well expressed and purified with the yield of 16.5 mg per liter of *E. coli* culture. After hydrophobic interaction chromatography, the mutant appeared to be approximately 99% pure, with a prominent protein band at around 24.3 kDa on SDS-PAGE (Figure 1). The minor protein band that moves faster than the major band on nonreducing SDS-PAGE was identified using nanoUPLC-ESI-q-TOF tandem MS (SYNAPT HDMS, Waters Co., Milford, MA, USA) with the previously published protocol [8]. It turned out to be a deletion version of the mutant Tnfaip8/Oxi-α not having *N*-terminal 36 amino acid residues (data not shown). The same deletion was also observed in the native form even though the purification was done at 277 K.

### 2.2. Protein Crystallization

**Figure 2.**
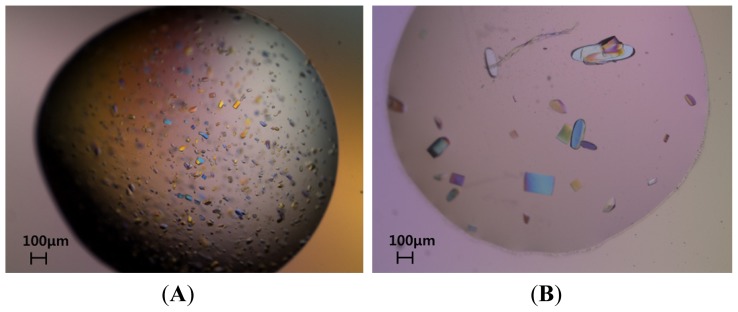
Crystals of C165S mutant Oxi-α. (**A**) Rice-shape crystals crystallized in 0.1 M sodium acetate pH 4.6, 1.0 M ammonium sulfate. The crystals grew to approximate dimensions of 0.10 × 0.10 × 0.02 mm in a week; (**B**) High-quality crystals crystallized in 0.1 M Bis-Tris propane pH 7.7, 1.2 M sodium citrate. These crystals grew to approximate dimensions of 0.50 × 0.25 × 0.10 mm in a week.

Although the protein solution was inhomogeneous, rice-shaped crystals of Tnfaip8/Oxi-α were obtained using ammonium sulfate as a precipitant (Figure 2A). It is noteworthy that 1.0 M ammonium sulfate was already included in the final concentrated protein solution. The improvement of crystal quality was achieved by addition of sodium citrate to the initial crystallization condition of 0.1 M sodium acetate pH 4.6, 1.0 M ammonium sulfate. Interestingly, the quality of crystals was gradually enhanced proportional to concentration of sodium citrate as described in the experimental section. Finally, high-quality crystals were obtained using a reservoir solution consisting of 0.1 M Bis-Tris propane pH 7.7, 1.2 M sodium citrate (Figure 2B). However, if ammonium sulfate was totally removed also from the concentrated protein solution, no crystal grew in the final crystallization condition.

### 2.3. X-ray Diffraction

The best crystal grew to dimensions of 0.50 × 0.25 × 0.10 mm within a week at 296 K (Figure 2B). When the crystal was exposed to synchrotron radiation under cryogenic conditions, diffraction limit was 1.8 Å resolution. This crystal belongs to space P2_1_2_1_2 and its Matthews coefficient [9] is 2.33 Å^3^·Da^−1^ when its asymmetric unit contains two Tnfaip8/Oxi-α protomers. These values are equivalent to solvent contents of 47.1%. The details of the data-collection statistics are presented in Table 2. Molecular replacement was performed by running PHASER [10] with the AutoMR wizard in PHENIX [11,12]. The crystal structure of Tnfaip8-like 2 (PDB entry 3f4m) was used as a search model. PHASER could place two protomers in the asymmetric with scores of RFZ (Rotation Function Z-score) = 13.4, TFZ (Translation Function Z-score) = 23.7 and LLG (Log Likelihood Gain) = 1489.0. A relatively good electron density map was obtained and the initial model of mutant Tnfaip8/Oxi-α was well established with the PHENIX AutoBuild wizard [13] with R- (Residual Factor) and R-free factors of 0.25 and 0.27, respectively. Full structure determination is in progress.

## 3. Experimental Section

### 3.1. Expression and Purification of Tnfaip8/Oxi-α

The cloning primers (Genotech, Daejeon, Korea) prepared for ligation-independent cloning (LIC) were 5′-GGCGGTGGTGGCGGCATGACCCTCATCGTTACCGGCG-3′ for the forward strand and 5′-GTTCTTCTCCTTTGCGCCCCTACAGCTGGCCGAACAGCCAAC-3′ for the reverse strand. The gene harboring murine Tnfaip8/Oxi-α (UniProt ID: Q921Z5) was amplified by PCR using 80 ng of the plasmid-cDNA [3] and 50 μM of primers with Deep Vent Polymerase (New England Biolabs, Inc., Beverly, MA, USA). The amplified LIC expression vector pB2 [14], a derivative of the pET21a vector (Novagen, Madison, WI, USA), was incubated with T4 DNA polymerase (New England Biolabs, Beverley, MA, USA) in the presence of 1 mM dATP at 310 K for 30 min followed by incubation at 343 K for 20 min. The amplified PCR product was prepared for vector insertion using the same protocol except for the presence of 1 mM dTTP instead of 1 mM dATP. The prepared insert was annealed into the pB2 vector, which expresses the cloned gene fused to a noncleavable *N*-terminal His_6_-tag, and was transformed into DH5α competent cells to obtain fusion clones. Clones were screened by plasmid DNA analysis and transformed into *E. coli* BL21 (DE3) for protein expression [15].

### 3.2. Growth of Cultures

*E. coli* BL21(DE3) transformed with the cloned vector containing the *Tnfaip8*/*Oxi-α* gene was grown on Luria-Bertani (LB) agar plates containing 150 μg·mL^−1^ ampicillin. Several colonies were picked and grown in capped test tubes with 10 mL LB broth containing 150 μg·mL^−1^ ampicillin. A cell stock of 0.85 mL culture and 0.15 mL glycerol was prepared and frozen at 193 K for use in a larger culture. The frozen cell stock was grown in 5 mL LB medium and diluted into 1000 mL fresh LB medium. The culture was incubated at 310 K with shaking until an OD_600_ of 0.6–0.8 was reached. At this point, expression of Tnfaip8/Oxi-α was induced using isopropyl β-D-1-thiogalactopyranoside at a final concentration of 0.5 mM. The culture was grown for a further 3 h at 310 K in a shaking incubator. Cells were harvested by centrifugation at 7650× *g* (6500 rev·min^−1^) for 10 min in a high-speed refrigerated centrifuge at 277 K.

### 3.3. Protein Purification

The cultured cell paste (5.32 g) was resuspended in 50 mL of buffer (0 mM Tris-HCl pH 8.0, 100 mM NaCl, 10 mM imidazole, 1 mM PMSF and 10 μg·mL^−1^ DNase I). The cell suspension was disrupted using a Digital Sonifier 450 (Branson Ultrasonics Co., Danbury, CT, USA). Cell debris was pelleted by centrifugation at 66,226× *g* (30,000 rev·min^−1^) for 30 min in a high-speed refrigerated ultra-centrifuge at 277 K. The supernatant was affinity-purified using a HisTrap column on an ÄKTA-explorer system (GE Healthcare, Piscataway, NJ, USA) at 277 K. The column was equilibrated with a buffer consisting of 50 mM Tris-HCl pH 8.0, 300 mM NaCl, 10 mM imidazole. The target protein was eluted with a buffer consisting of 50 mM Tris-HCl pH 8.0, 100 mM NaCl with a gradient from 10 to 500 mM imidazole. The pooled Oxi-α was purified by ion-exchange chromatography using a 5 mL Hi-Trap S column (GE Healthcare, Piscataway, NJ, USA) equilibrated with a buffer consisting of 10 mM Sodium phosphate pH 6.0. Tnfaip8/Oxi-α was further purified by hydrophobic chromatography using a 5 mL Hi-Trap phenyl HP column (GE Healthcare, Piscataway, NJ, USA) equilibrated with a buffer consisting of 1 M ammonium sulfate, 50 mM Sodium phosphate pH 7.0. The protein was included in a flowthrough. SDS-PAGE showed one band around 23 kDa corresponding to the molecular weight of His_6_-tagged Tnfaip8/Oxi-α. The purified protein contained a noncleavable *N*-terminal His_6_-tag followed by five glycine residues (MHHHHHHGGGGG) and was concentrated to 2~3 mg·mL^−1^ (using an extinction coefficient of 0.26 M^−1^·cm^−1^ at 280 nm) for crystallization in a buffer consisting of 1 M ammonium sulfate, 50 mM sodium phosphate pH 7.0. Unfortunately, the concentrated protein solution was continuously precipitated even in the presence of 20% glycerol.

### 3.4. Site-Directed Mutagenesis

The expression plasmid pB2-Oxi-α was used as a template for mutagenesis. The expression plasmid including the C165S mutant gene was synthesized (AbFrontier, Seoul, Korea) and was transformed into *E. coli* BL21(DE3) for expression [14]. The mutant protein was purified using the same protocol mentioned above for the wild type. The purified mutant contained a noncleavable *N*-terminal His_6_-tag followed by five glycine residues (MHHHHHHGGGGG) and was concentrated to 16.5 mg·mL^−1^ for crystallization in a buffer consisting of 1 M ammonium sulfate, 50 mM sodium phosphate pH 7.0.

### 3.5. Crystallization of Mutant Tnfaip8/Oxi-α

Screening for crystallization conditions was performed at room temperature with several commercial screens such as Crystal Screen I, II and Index Screen kits from Hampton Research (Laguna Niguel, CA, USA). A Hydra-Plu-One crystallization robot (Matrix Technologies, Hudson, NH, USA) was used to set up the screens using the sitting-drop vapour-diffusion method in a 96-well Intelli-Plate (Art Robbins Instrument, Salt Lake City, UT, USA). Sitting drops were made by mixing 0.2 μL protein solution (16.5 mg·mL^−1^) with 0.2 μL reservoir solution and were equilibrated against 50 μL reservoir solution. A VDX48 plate (Hampton Research, Laguna Niguel, CA, USA) was used to optimize the crystallization conditions using hanging drops produced by mixing 0.8 μL protein solution and 0.8 μL reservoir solution and equilibrated against 200 μL reservoir solution. The initial crystals grown in the crystallization solution of 0.1 M sodium acetate pH 4.6, 1.0 M ammonium sulfate were rice-shaped (Figure 2A). This condition was further optimized using the Additive kit from Hampton Research. The improvement of crystal size was achieved by addition of sodium citrate. Interestingly, the size of crystals became gradually larger and the edge of crystals became clearer proportional to concentration of sodium citrate. Finally, high-quality crystals were grown within a week at 296 K with the crystallization solution consisted of 0.1 M Bis-Tris propane pH 7.7, 1.2 M sodium citrate (Figure 2B).

### 3.6. Data Collection and Reduction

Before flash-cooling in liquid nitrogen, crystals were soaked in LV CryoOil (MiTeGen, Ithaca, NY, USA). X-ray diffraction data were collected at a single wavelength (λ = 0.97933 Å) with one crystal on beamline 7A at Pohang Light Source using a Quantum 4 CCD detector (Area Detector Systems Co., Poway, CA, USA) placed 300 mm from the sample. The oscillation range per image was 1.0°, with 2 s exposures, and 180 oscillation images were collected with no overlap between two contiguous images. X-ray diffraction data were processed and scaled using DENZO and SCALEPACK from the HKL-2000 program suite [16].

## 4. Conclusions

The cellular and molecular roles of Tnfaip8/Oxi-α family members in the cells have not yet been fully understood. Therefore, we performed crystallographic and biochemical studies. In the previous study, we identified a Tnfaip8/Oxi-α linked protective role for dopaminergic cells against OS-induced cell death [4]. However, crystallization with native Tnfaip8/Oxi-α was not successful due to heavy precipitation presumably caused by non-specific disulfide bond formation. In this study, we have overcome the problem by mutating Cys165 to Ser165. High-quality crystals with diffraction patterns extending up to 1.8 Å resolution were obtained by a gradual increment of sodium citrate concentration. The diffraction data could be used for structure solution. A further biochemical study based on the initial crystal structure is ongoing.

## Figures and Tables

**Table 1.** Tnfaip8/Oxi-α family members.

Family members	Tnfaip8/Oxi-α	Tnfaip8l1/Oxi-β	Tnfaip8l2/Oxi-γ	Tnfaip8l3/Oxi-δ
Sequence identity with Tnfaip8/Oxi-α	100%	54.4%	53.9%	54.4%

**Table 2.** Data collection statistics for the mutant Oxi-α crystal. The values in parentheses are for the highest resolution shell.

X-ray source	Pohang Accelerator Laboratory (PAL)
X-ray wavelength (Å)	0.97933
Temperature (K)	100
Space group	P2_1_2_1_2
Unit cell parameter	
-*a* (Å)	66.9
-*b* (Å)	72.3
-*c* (Å)	93.5
Volume fraction of solvent (%)	47.1
V_m_ (Å^3^/Dalton)	2.33
Resolution range (Å)	50.0–1.8 (1.83–1.80)
Total number of observed reflections	181684
Multiplicity	4.4 (4.5)
Unique reflections	41052 (2090)
R_sym_ † (%)	7.7 (46.1)
Data completeness (%)	95.7 (99.1)
Average I/σ (I)	16.4 (2.9)

† *R*_sym_ = ∑_hkl_ ∑_i_|*I*_hkl, i_ –〈*I*〉_hkl_|/∑_hkl_ ∑_i_ *I*_hkl, i_, where *I*_hkl, i_ is the intensity of the ith observation of the unique reflection hkl and 〈I〉_hkl_ is the mean of the intensities of all observations of reflection hkl.
